# Expanded GEP-NET organoid culture for personalized therapy evaluation

**DOI:** 10.1126/sciadv.aea4296

**Published:** 2026-07-24

**Authors:** Steven D. Forsythe, Srujana V. Yellapragada, Tracey Pu, Darryl Nousome, Samarth Mathur, Dilara Akbulut, Stephen G. Andrews, Kamala A. Kenny, Martha Quezado, Margaret C. Cam, Jaydira Del Rivero, Serguei Kozlov, Jonathan M. Hernandez, Naris Nilubol, James P. Madigan, Samira M. Sadowski

**Affiliations:** ^1^ Neuroendocrine Cancer Therapy Section, National Cancer Institute, National Institutes of Health, Bethesda, MD 20892, USA.; ^2^ Surgical Oncology Program, National Cancer Institute, National Institutes of Health, Bethesda, MD 20892, USA.; ^3^ CCR Collaborative Bioinformatics Resource, Office of Science and Technology Resources, National Cancer Institute, National Institutes of Health, Bethesda, MD 20892, USA.; ^4^ Advanced Biomedical Computational Science, Frederick National Laboratory for Cancer Research, Frederick, MD 21702, USA.; ^5^ Laboratory of Pathology, Center for Cancer Research, National Cancer Institute, National Institutes of Health, Bethesda, MD 20892, USA.; ^6^ Center for Advanced Preclinical Research, Frederick National Laboratory for Cancer Research, Center for Cancer Research, National Cancer Institute, Frederick, MD 21702, USA.; ^7^ Developmental Therapeutics Branch, National Cancer Institute, National Institutes of Health, Bethesda, MD 20892, USA.

## Abstract

Gastro-entero-pancreatic neuroendocrine tumors (GEP-NETs) are a rare subset of cancers with increasing incidence. Due to their slow growth and lack of targetable mutations, the identification of effective treatments remains limited. One reason behind this stagnation is the lack of applicable, accurate study models. One solution is patient tumor organoids (PTOs) that maintain tumor characteristics and can scale for high throughput assays. In this study, PTOs were generated from 35 tumors of pancreatic, small intestinal, and gastric origin, obtained from 17 patients. Important subtypes including hormone functional and *MEN1*/*VHL* mutant GEP-NETs are represented, with each demonstrating growth in culture while maintaining GEP-NET immunohistochemistry and genomic characteristics. Half of G2/G3 tumors (10 of 20) could be cultured past passage 6, whereas G1 tumors (*n* = 15) were capable of growth until passage 4. Therapeutic targeting of the PTOs displayed both tissue-origin and grade-based response to standard of care and investigational therapies while maintaining patient tumor sensitivity and resistance. Last, a successful PTO xenograft model was developed from one PTO line. This study describes GEP-NET organoid development that demonstrates feasibility for expansion, enabling their use for translational investigations.

## Teaser

Patient-derived GEP-NET organoids are maintained for several passages and show clinically relevant therapeutic responses.

## INTRODUCTION

Gastro-entero-pancreatic neuroendocrine tumors (GEP-NETs) are a rare subgroup of endocrine cancers arising throughout the gastrointestinal tract, including the pancreas, stomach, and bowel. They have a combined incidence rate of 5.45 cases per 100,000, which has risen fivefold during the past four decades (*1*, *2*). These tumors can be divided into functional and nonfunctional (NF) subtypes based on hormone secretion and clinical symptoms, with ∼2 of 3 classified as NF (*1*). Further stratification within GEP-NETs is based on grading and cell differentiation, with percent ki67-positive cells as an index of proliferation rate: well-differentiated grade 1 (G1; <3%), grade 2 (G2; 3 to 20%), and grade 3 (G3; >20%). Treatment options and sequencing for now approved therapies are often based on multidisciplinary evaluation taking into account functional status, metastatic disease burden, and tumor grade, which often fall short for both metastatic disease and higher-grade tumors with poor survival outcomes (*3*). The tumor mutational burden (TMB) of GEP-NETs has been reported as one of the lowest among tumor types, which reveals few targetable mutations (*4*). Of the genes commonly found mutated, many are genetically linked tumor suppressor genes including *MEN1*, *DAXX*, *P53*, *CDKN1B*, and *ATRX*, rather than targetable driver mutations such as *RAS* or *MYC* (*5*, *6*). This also includes low penetrance of these driver mutations, with the most common only found in ∼20% of patients with GEP-NET. In addition, expression of immune-mediated efficacy markers, such as PD-L1, are low and limited to high-grade tumors, which have shown low response toward current immune checkpoint inhibitors (*7*, *8*). Last, while the expression of somatostatin receptors on the surface of some GEP-NETs has led to marked improvements in receptor-targeted tumor detection and treatment modalities, this expression is inversely correlated to tumor grade, necessitating new therapy options to navigate the intricacies of subtype and grade-based expression (*9*–*12*).

One of the difficulties in identifying effective therapies for GEP-NETs is a lack of reproducible disease models for in vitro and in vivo experimentation. Efforts to establish models of neuroendocrine cancers have faced a long and challenging road (*13*, *14*). Early cell lines closely represent neuroendocrine carcinomas (NECs), which contain a different mutational and growth profile and can often suggest ineffective treatments (*15*, *16*). Animal models have shown marked differences in tumor biology or face difficulties in establishment, especially when modeling complex conditions such as hereditary *MEN1* loss. In addition, success rates for patient-derived xenografts (PDXs) are reported to be around 10%, making this a low success and expensive endeavor. Recently, three-dimensional nonanimal models, including patient tumor organoids (PTOs), have moved to the forefront of cancer modeling for a variety of cancers. PTOs demonstrate many advantages over both cell lines and animal models and represent the middle ground between the two regarding accuracy, throughput, and cost. In addition, PTOs can be used for rapid and reproducible testing to predict patient response to a range of treatments, including chemotherapy, targeted therapy, and immunotherapy (*17*, *18*). Efforts to model NETs in three-dimensional culture have been ongoing, with substantial strides shown in therapy testing (*8*, *19*–*22*). However, there remain several shortcomings, such as low rates of growth for more than a single passage. This is likely due to the indolent nature of these tumors. This remains a serious issue as experiments requiring large amounts of organoids for replicative studies, including CRISPR-Cas9 gene editing, ultrahigh-throughput therapy screening, and signal transduction studies, remain difficult to achieve for GEP-NETs. While PTOs demonstrate promise, there remains a demand for long-term, reproducible organoids for use in replicate studies.

To address this research gap, we developed a culture method for PTOs derived from well-differentiated GEP-NET tumors. Our team demonstrated the ability to culture and grow GEP-NETs from a variety of tissue origins and grades over several passages. We hypothesized that GEP-NET PTOs would demonstrate clinically relevant therapeutic responses and maintain fidelity. These findings demonstrate the potential in personalized medicine for future therapeutic development.

## RESULTS

### Establishment of GEP-NET organoid culture

**Fig. 1.**
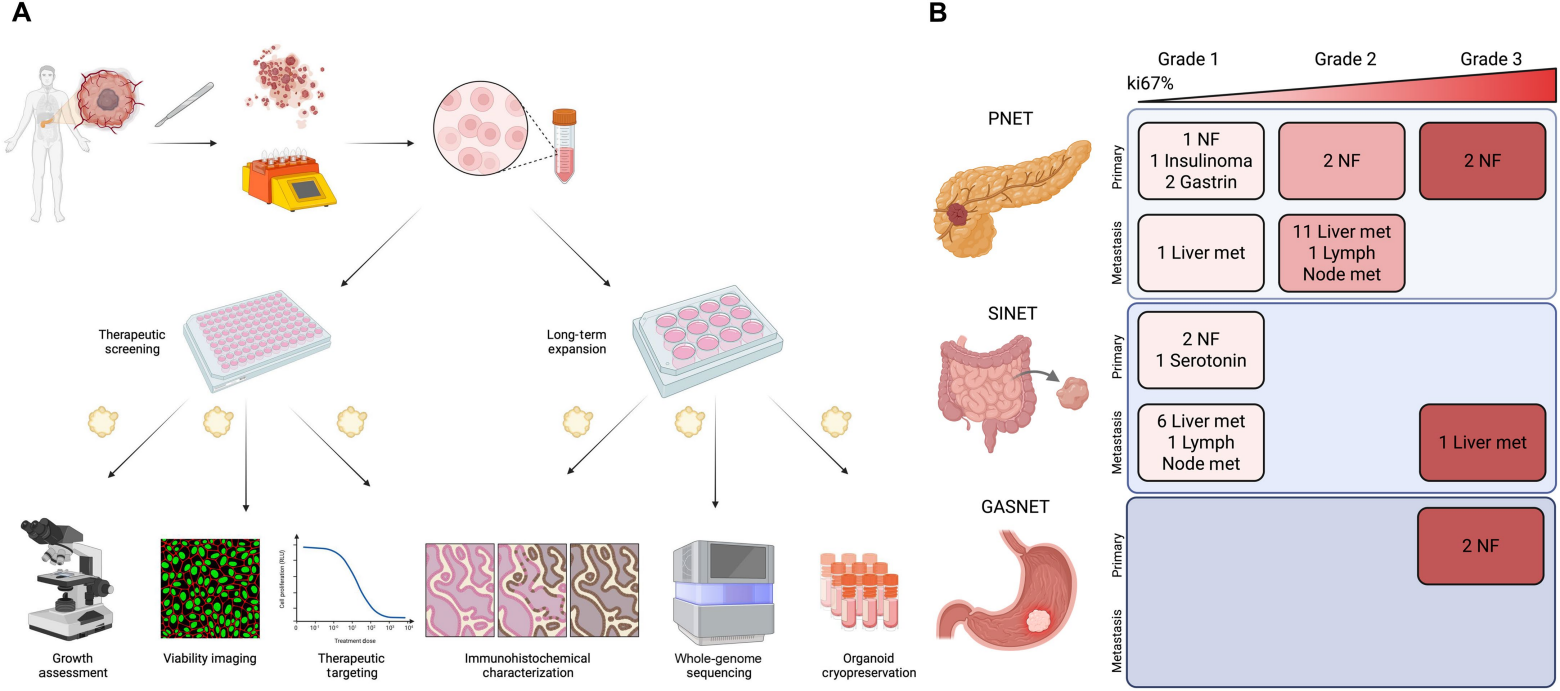
Generation of GEP-NET PTOs from patient tissues. (**A**) Flowchart of experimental workflow. Briefly, patient tumor cells are isolated from tissue and used in both short-term therapeutic screening and long-term organoid culture for expansion. (**B**) Patient tumors organized by origin [pancreatic neuroendocrine tumor (PNET), small intestinal neuroendocrine tumor (SINET), and gastric neuroendocrine tumor (GASNET)], grade, functional status [nonfunctional (NF)], and by primary versus metastasis (m). RLU, relative luminescent units. Created in BioRender. S. D. Forsythe (2026), https://BioRender.com/buqxw6v (A) and https://BioRender.com/dei49i4 (B).

From March 2023 to December 2023, 35 tumors from 17 patients were procured for organoid culture (Fig. 1 and table S1). Twenty-two tumors were procured from 11 patients diagnosed with pancreatic neuroendocrine tumors (PNETs) (nine primaries, 12 liver metastases, and one lymph node metastasis), 11 tumors were procured from five patients with small intestinal neuroendocrine tumors (SINETs) (three primaries, seven liver metastases, and one lymph node metastasis), and two primary tumors were procured from one patient diagnosed with a gastric neuroendocrine tumor (GASNET). Five patients were diagnosed with functional tumors, including one patient with pancreatic insulinoma, three patients with serotonin-producing small intestinal tumor, and one patient with gastrin-producing pancreatic tumor. Six patients were previously diagnosed with *MEN1* germline mutations, two patients with *VHL* germline mutations, and three patients with somatic *MEN1* mutations. Fifteen tumors were diagnosed as G1 from seven patients, 15 tumors were diagnosed as G2 from five patients, and 5 tumors were diagnosed as G3 from four patients.

### PTOs retain growth and viability over multiple passages

**Fig. 2.**
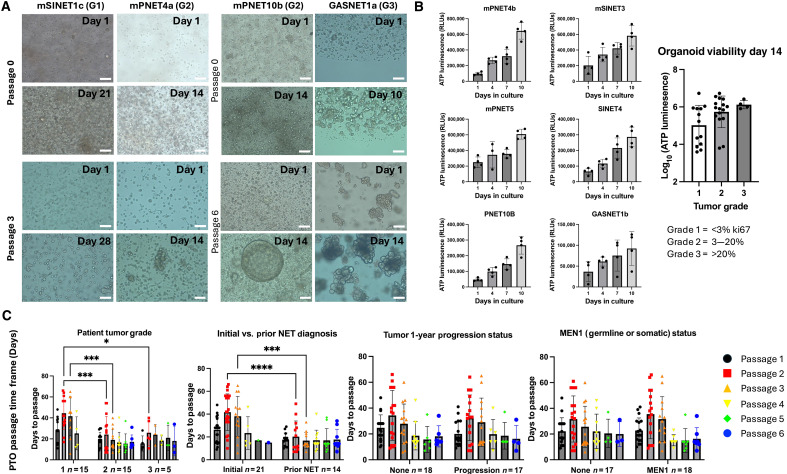
GEP-NET organoids demonstrate grade-based growth and can be propagated for several passages. (**A**) Bright-field light microscopy images of mSINET1c [grade (G1)], SINET4 (G2), mPNET10b (G2), and GASNET1a (G3) demonstrate growth based on grade. Scale bars, 100 μm. (**B**) GEP-NET organoids demonstrated consistent growth over 14 days, with higher-grade PTOs showing higher average viability values using CellTiter Glo assay. *n* = 4 technical replicates for individual tumors and biological for organoid viability at 14 days. (**C**) PTOs demonstrate increased time to the next passage when separated by grade or by status as initial or recurrent tumor (prior GEP-NET), while tumor origin and status as primary or metastasis did not. All replicates are biological and comparisons are made using Student’s *t* test between time points.

The utility of PTOs for long-term study is dependent on their ability to be reproducibly and accurately cultured over multiple passages. To ensure that organoids were capable of long-term culture, PTOs were cultured until the formation of large clusters of cells (Fig. 2A). PTOs were cultured until lack of proliferation exceeding 60 days was noted, or until fibroblast populations were observed to overtake tumor cell cultures (table S2). In addition to imaging organoids in long-term culture, we tested the organoids at multiple time points during the first 14 days of culture to assess growth and ensure that PTOs were viable and metabolically active for therapeutic screening. PTOs demonstrated visual and metabolic growth and expansion during this time frame regardless of grade, and formation of organoid clusters was observable within 2 weeks as determined by light microscopy and CellTiter Glo 3D assay (Fig. 2B). The average viability of PTOs at day 14 trended toward statistical significance, with higher-grade PTOs demonstrating increased viability values when compared to lower-grade PTOs (*P* = 0.06). Organoids demonstrated variable growth between passages when stratified by grade (*P* < 0.001), by initial versus prior NET tumor patient status at time of surgery (*P* = 0.01), by tumor progression within 1 year of surgery (*P* < 0.001), and by MEN1 mutation status (*P* < 0.001); of note, growth characteristics as defined by time to passage was stable between passage 1 and 2, followed by a general decrease in subsequent passages (Fig. 2C and table S2). There were no statistically significant differences in the number of days to the next passage between organoids from GEP-NET primary tumor origins, patients treated with somatostatin analog neoadjuvant, or between primary and metastatic lesions at any passage. However, required culture time frame between passages was linked to tumor grade, with G1 tumors demonstrating slower progression to passage 2 when compared to G2 (44.5 versus 24.2 days, *P* < 0.001) and G3 (44.5 versus 25.8 days, *P* = 0.02) and slower progression to passage 3 when compared to G2 (41.6 versus 19 days, *P* < 0.001), respectively. None of the 15 G1 organoid lines progressed past passage 4, whereas 47% (7 of 15) of G2 lines and 60% (3 of 5) of G3 lines were able to reach passage 6. In addition, tumors from patients with a prior NET diagnosis demonstrated faster in vitro growth kinetics than those that were at initial presentation (*P* = 0.01), particularly at passage 2 (41.7 days versus 20.3, *P* < 0.001) and at passage 3 (38.0 days versus 16.8, *P* < 0.001), respectively. Only one tumor at initial presentation, the G3 tumor PNET6, reached passage 6 (1 of 21, 4.76%) compared to 10 of the 14 (71.4%) recurrent tumors.

### PTOs resemble NET morphology

**Fig. 3.**
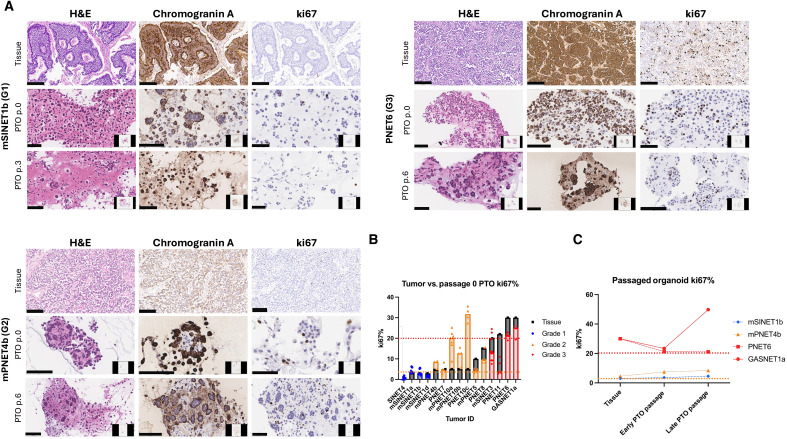
GEP-NET organoids retain original patient tumor tissue characteristics and maintain tumor grade. (**A**) Patient PTOs match neuroendocrine characteristics [hematoxylin and eosin (H&E) and chromogranin A] and maintain tumor grade and proliferative capacity (ki67). Scale bars, 100 μm for all patient tissue sections,100 μm for PTO H&E and chromogranin A, and 50 μm for PTO ki67. (**B**) PTOs match tumor grade (%ki67^+^ cells) in 10 of the 15 cultures. All replicates technical replicates. (**C**) PTOs maintain tumor grade after several passages.

Neuroendocrine immunohistochemistry (IHC) markers were used to assess neuroendocrine phenotype of the organoids. PTOs demonstrated expression of chromogranin A, as observed by an expert pathologist (Fig. 3A). Similarly, PTOs also expressed neuroendocrine marker synaptophysin and maintained surface expression of somatostatin receptor 2 (SSTR2) (fig. S1). These results matched comparisons of IHC performed on tumor tissue sections and were maintained during multiple organoid culture passages. A comparison of the ki67 mitotic proliferation rate between tumor organoids and the percentages reported by pathology on tumor tissues revealed that tumor organoids matched their parent tumor tissue grade in 10 of the 15 (67%) comparisons (Fig. 3B). Two G3 tumor tissues produced G2 organoids (PNET11, pathology reported 20% versus 3.7% ki67 mitotic rate in tumor organoid, and mSINET3, pathology reported >20% versus 13% in tumor organoid). Organoid mSINET3 had focal regions of >20% ki67, presenting potential issues in sampling for the original tissue. Two lesions from SINET1 generated G2 organoids (*a* = 4.1% and *c* = 3.6%), with individual regions of interest located on both sides of the G1/G2 cutoff while another lesion (*d*, ki67% = 1.6%) was scored as G1. One G2 tumor tissue generated a G3 organoid (mPNET10c, 5% pathology reported versus 32% in tumor organoid). As most of the lesions presented higher than expected ki67% for PNET10, it is possible sampling also provided higher growth subpopulations not seen in the original tumor pathology. Organoids were also compared after passaging and demonstrated similar grades to both the original tumor tissue and original PTO passage up to passage 3 for G1 and passage 6 for G1/G2 (Fig. 3C).

### PTOs are representative of NET tissue mutational profile

**Fig. 4.**
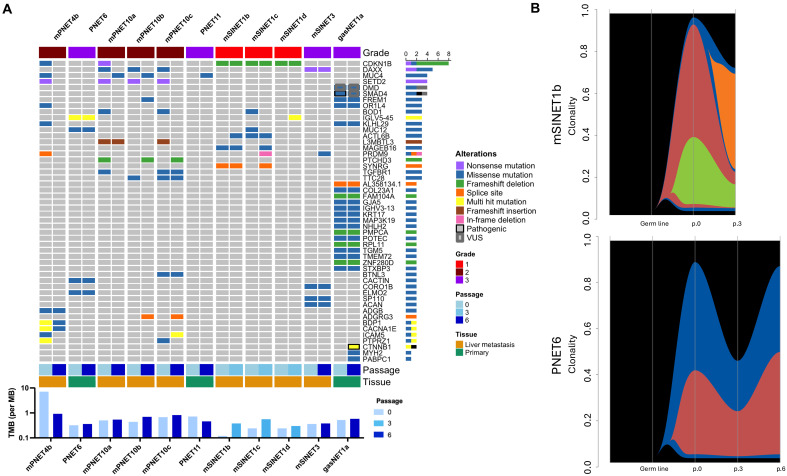
GEP-NET organoids retain tumor tissue mutations and demonstrate maintenance of clonal subpopulations. (**A**) Oncoplot of tumor tissue mutations compared to germline data demonstrates diverse mutations, with maintenance of TMB between PTO passages graphed below. (**B**) River plot of clonal populations illustrates maintenance of subpopulations between representative patient germ line, tumor tissue, and derived PTO. Color-matched listing of respective subpopulation mutations by name and type, with black representing germ line.

To confirm the presence of parent tumor tissue mutations in our PTO lines, we used whole-genome sequencing of PTOs at passages 0 and either 3 or 6, depending on the final passage reached, and compared to patient germline DNA from blood to identify tumor-specific mutations (Fig. 4A). A mutational change of one or fewer mutations was observed in 5 of the 11 (45%) tumor/organoid pairs sequenced, with the average number of changes at 3.8 per organoid line. The most frequently observed mutations in PTOs were *CDKN1B* alterations (5 of 11, 45%), followed by *DAXX*, *MUC4*, *SETD2*, and *DMD*. Regarding mutational changes between passaging, the most observed acquired mutation was Mucin 4 (*MUC4*) (4 of 11, 36%), whereas *SETD2* was lost in 4 of the 11 tumor/PTO pairs. TMB was observed to increase in 9 of 11 (82%) and decrease in 2 of 11 (18%) PTOs, with only one patient experiencing a change of greater than 10-fold (mPNET4b). Analysis of patient tumors from germline to passaged organoids demonstrated maintenance of clonal population, with some shifts in population noted and in line with expected clonal selection (Fig. 4B). Each PTO group maintained multiple clonal subpopulations, with evolution of tumor mutations in organoids further distinguishing these cellular colonies.

### Therapeutic interrogation demonstrates origin, grade, and personalized PTO treatment responses

**Fig. 5.**
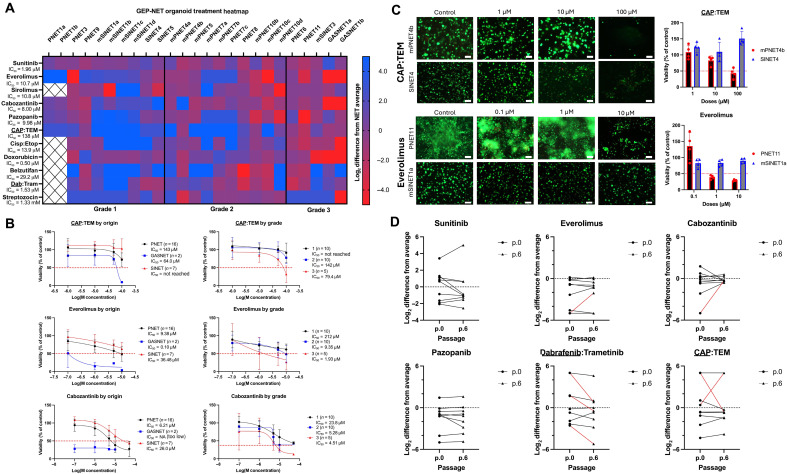
GEP-NET PTOs are instrumental in demonstrating therapeutic efficacy. (**A**) Heatmap of all GEP-NET PTOs for treatment. Values below zero (red) indicate increased sensitivity toward therapy, whereas higher than zero (blue) indicate resistance when compared to the overall GEP-NET IC_50_ across all PTOs (NET average), with boxes marked with a “X” not performed. Each box composed of technical replicates. (**B**) Separation of GEP-NET PTO response by tissue origin and grade shows variability in tumor response for cabozantinib, everolimus, and CAP:TEM. Each point composed of biological replicates. (**C**) LIVE/DEAD images of treatment sensitive (mSINET3, mPNET4b, and PNET11) and treatment resistant (mPNET3a, SINET4, and mSINET1a) PTOs show viable cells (green) and dead nuclei (red) following therapy. Scale bars, 100 μm. Red dotted line on graph is equal to 50% cell death. (**D**) Passaged PTOs demonstrate similar response to therapies when compared to the original PTOs, with red group indicating >2-log IC_50_ difference when compared to original PTO. Each point is a technical replicate set of *n* = 4.

To demonstrate the utility of PTOs in therapeutic drug screening applications, we used a panel of therapies that are both in current standard of care practice and under investigation (Fig. 5A). Therapies included multiple agents targeting the same pathways, including mammalian target of rapamycin (mTOR) inhibitors and platelet-derived growth factor receptor/vascular endothelial growth factor receptor inhibitors. PNET organoids demonstrated superior response to cabozantinib (*P* = 0.009), pazopanib (*P* = 0.04), and belzutifan (*P* = 0.02) when compared to SINET organoids (Fig. 5, B and C, and fig. S2A). As only one patient provided gastric NET tissue, we did not compare treatment responses to either PNET or SINET. We observed that several therapies were more efficacious in higher-grade tumors (G2/G3) compared to lower-grade tumors (G1) (fig. S2B). This was especially notable when comparing cytotoxic chemotherapies. G1 demonstrated lower treatment response compared to G2 when treated with streptozocin (*P* < 0.01) and a lower treatment response when compared to G3 when treated with everolimus (*P* = 0.01), cabozantinib (*P* = 0.03), capecitabine:temozolomide (CAP:TEM) (*P* < 0.001), cisplatin:etoposide (*P* = 0.01), and doxorubicin (*P* < 0.05). G2 demonstrated an inferior response compared to G3 when treated with CAP:TEM (*P* = 0.002), cisplatin:etoposide (*P* = 0.003), and doxorubicin (*P* = 0.04). PTOs derived from NF tumors were more sensitive to therapies cabozantinib (*P* < 0.05), everolimus (*P* < 0.05), dabrafenib:trametinib combination therapy (*P* < 0.05), and belzutifan (*P* < 0.05). PTOs from patients with prior NETs were more sensitive to cabozantinib (*P* < 0.05), everolimus (*P* < 0.05), CAP:TEM (*P* < 0.05), belzutifan (*P* < 0.05), and doxorubicin (*P* < 0.05). MEN1 mutation status (either germline or somatic) conferred increased therapeutic sensitivity toward cabozantinib (*P* < 0.01), everolimus (*P* < 0.05), CAP:TEM (*P* < 0.05), pazopanib (*P* < 0.05), and sirolimus (*P* < 0.05). One-year patient recurrence status demonstrated increased sensitivity toward cabozantib (*P* < 0.05), whereas primary tumor-derived PTOs were more sensitive toward doxorubicin (*P* < 0.05) compared to metastatic PTOs. There was no statistical significance for treatments in PTOs with SSTR neoadjuvant therapy compared to no treatment. To assess whether PTO treatment responses drifted over time, we assessed their viability at passage 0 and later passage 3/6 (Fig. 5D). When comparing therapy responses, ∼64% (34 of 54) of later passage PTOs were within onefold log_2_ difference of the original passage 0 PTO sets and 83% (45 of the 54 treatments) were within a twofold log_2_ difference of the original therapy response when analyzing six therapies across nine PTO sets, demonstrating consistent therapy sensitivity for each PTO set across passages.

Two patients in this dataset received non-SSTR2 analog neoadjuvant therapy and were compared to their matched PTOs (Fig. 5A and table S1). The patient with PNET9 received two cycles of CAP:TEM within 1 year of surgery, whereas the patient with GASNET1 received multiple cycles of sunitinib over the course of several years until disease progression, signaling acquired resistance. PTOs of PNET9 demonstrated modest response to CAP:TEM (66% viability at 100 μM), a response typical of G1 tumors to this regimen, with LIVE/DEAD imaging illustrating this resistance (fig. S3). PTO of GASNET1a demonstrated high resistance toward sunitinib therapy, similar to original patient tumors, with a median inhibitory concentration (IC_50_; 15.5 μM) 2.89 log_2_ fold difference from the GEP-NET average. Another patient (PNET8) with a von Hippel-Lindau (VHL) mutation would be a potential candidate for belzutifan therapy, a HIF2 alpha inhibitor now approved for VHL-associated PNETs (*23*). Substantial efficacy was observed in the PTOs from this one patient (>−5 log_2_ fold change when compared to GEP-NET average). This individual response demonstrates a potential therapeutic option for this patient should tumors recur, pending further validation in a larger cohort.

### PTOs can be used to develop stable PTOX

**Fig. 6.**
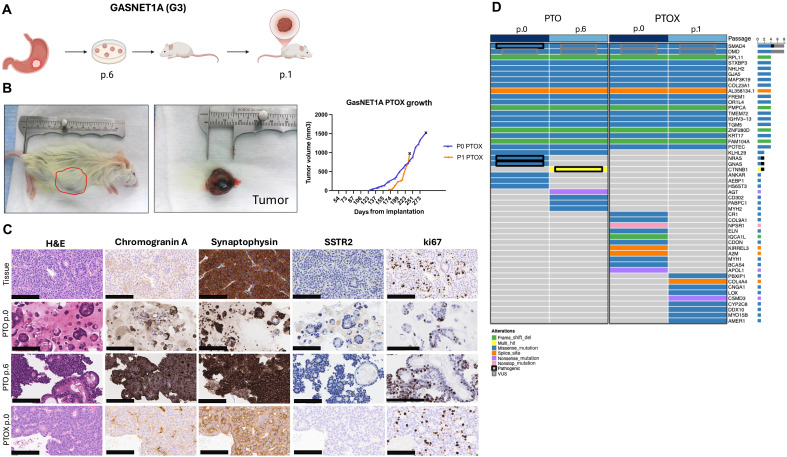
Development of a PTOX model from GASNET1A. (**A**) Schematic describing the implantation of GASNET1. PTOs from passage 6 were isolated and flank implanted into mice and allowed to develop PTOX models. (**B**) Visual confirmation of tumor growth and image of resected tumor tissue, with growth chart of PTOX at passages 1 and 2. (**C**) IHC comparing original tissue, PTOs at passages 0 and 6, and PTOX at passage 1 demonstrates similar cellular morphology, expression of chromogranin and synaptophysin, lack of SSTR2 expression, and maintenance of G3 status. Scale bars, 100 μm for all patient tissue and PTO sections. (**D**) Oncoplot comparing PTOs at passages 0 and 6 compared to PTOX at passages 0 and 1. Created in BioRender. S. D. Forsythe (2026), https://BioRender.com/ukqeuks.

Despite their versatility, PTOs still face challenges in simulating systemic organism-level responses and predicting the nontumor side effects of therapeutics. To address these issues, we subcutaneously implanted one PTO model (GASNET1A at p.6) into the flank of immune-compromised nonobese diabetic (NOD) scid gamma (NSG) mice (*n* = 2) to establish a PTO xenograft (PTOX) model (Fig. 6A). Following 123 days, palpable tumors were detected on one mouse, and, at 273 days, these tumors were harvested and reimplanted into two mice; at the second passage, the tumor was palpable at day 171 on one mouse and harvested at day 235 (Fig. 6B). Tumors stained positive for chromogranin A, strongly positive for synaptophysin with a decrease in the PTOX p.0, had no expression of SSTR2 and maintained G3 status by ki67 rate as observed in the original tumor tissue and the parent PTO population (Fig. 6C). IDEXX Laboratories' short tandem repeat (STR) sequencing (100% tumor identity match) and genetic fingerprinting analysis [logarithm of the odds (LOD) score of >10^72^] confirmed with high certainty that all samples were from the same human source (tables S3 and S4). Genomic analysis demonstrated maintenance of highly prevalent mutated genes including *SMAD4*, *DMD*, and *RPL11* across PTO and PTOX models (Fig. 6D). Last, diverse tumor cell populations were observed and maintained at several points including the transition of organoid culture to PTOX and between passages (fig. S4).

## DISCUSSION

GEP-NETs remain an understudied and poorly understood cancer group. With a paucity of available models, research efforts to identify effective treatments have been limited. Several major milestones must be achieved to consistently use suitable GEP-NET models: high rate of establishment, maintenance of tumor characteristics (including neuroendocrine markers and grade-based ki67 proliferation index), ability to test therapy response, and the reproducibility of results over multiple passages. This study pioneers the establishment and testing of pharmacologic agents of interest in patient-derived GEP-NET organoids within long-term culture, covering multiple primary tissue origins, metastatic sites, and tumor grade.

This study described the consistent production and usage of PTOs from GEP-NETs of multiple grades and tissue origins. Previous reports described low success for the generation of PTOs, often limited to G2/G3 tumors or NECs. Success rates for establishment past passage 1 have been limited to below 25% and only from G2/G3 tumors (*20*). In our study, steady growth was observed among almost all organoid cultures for the first 2 weeks, with increasingly large organoids developing in cultures across grades and tissue origins. Half of all G2/G3 tumors reached passage 6 (10 of 20, 50%). Growth of this degree and duration would allow for the generation of PTOs for usage as promising investigative tools, including CRISPR-Cas9 gene editing, large-scale high-throughput screening, and volumes suitable for PDXs. Our PTOs also maintained tumor characteristics including chromogranin A expression, cellular morphology, and tumor grade. While there were some changes in tumor grade observed among the PTOs, these were based on comparisons to the original tumor tissue pathology on a separate surgical biopsy, creating potential intratumoral sampling differences.

Retaining tumor mutations across passages is key toward preserving a model’s utility for future research. Balancing successful establishment versus accuracy allows for reproducible investigation of a model. Our data are consistent with prior studies, which reported 66.7 to 100% maintenance of tumor mutations, albeit these studies were performed with fewer PTOs and on short-term cultures (*21*, *22*). Clonal analysis of PTOs demonstrated the presence and maintenance of tumor subpopulations, with subclones surviving for several passages. *CDKN1B* mutations were the most common in both PNET and SINET PTO lines. *CDKN1B* encodes the protein p27 with mutations in this gene, resulting in loss of cell-cycle checkpoint control. It has been described in a wide range of cancers, including NETs (*24*). Each PTO showed some shift from their early passage to later passage, notably in acquisition of mutations in *MUC4* and loss of *SETD2* and *DAXX*. *MUC4* has been described to be mutated in both PDAC and PNET tumors and is overexpressed when compared to normal pancreatic tissue (*25*, *26*). *MUC4* has demonstrated a role in tumor progression and is correlated with worse tumor survival for many cancers, with direct targeting resulting in the cessation of cellular growth. There have been efforts to develop therapies targeting MUC4, although none have been approved for clinical use (*25*). SETD2 is involved in histone modification and is shown to harbor tumor suppressor characteristics. Inactivating mutations in this gene lead to cancer development in the lung, gastrointestinal tract, and pancreas (*27*). *DAXX* mutations are often found in GEP-NET tumors, playing a role in dysregulated p53 chromatin binding, highlighting its likely role in tumor suppression (*28*). While our study demonstrates a loss in both *SETD2* and *DAXX* in long-term PTO culture, we did not observe a concomitant shift in tumor treatment sensitivity in more than 80% of PTOs tested, indicating that tumor treatment sensitivity was unaffected by the shift in these mutations.

PTOs are an exceptionally powerful tool for predicting treatment responses, offering unprecedented opportunities to revolutionize future health care strategies. Considering tumor cell population maintenance, rapid development, and quick treatment turnaround, results can be obtained within weeks and before decisions based on potential adjuvant therapy need to be finalized. In other cancers, several groups have published clinical correlation studies when comparing the response of chemotherapy and immunotherapy in PTOs (*17*, *18*). Reliable reproduction of tumor biology as indicated by maintenance of tumor grade classification allows for sophisticated therapeutic investigation. The Commonwealth Neuroendocrine Tumor Collaboration and North American Neuroendocrine Tumor Society consensus guidelines recommend tumor grade and ki67 utilization as a prognostic and treatment-informing biomarker as a higher ki67 index may predict a shorter duration of response to somatostatin analog therapy (*29*). When comparing treatments based on tumor grades, our results match the current clinical paradigm in treating GEP-NETs, which recommends the usage of chemotherapy primarily in G3 NETs and only in select cases of G1/G2 NETs (*30*, *31*). This is due to the highly proliferative nature of these advanced grade tumors, which allows for increased efficacy due to higher cellular division and drug uptake. Other factors including functional status and prior NET resection status may also be linked to tumor aggressiveness and their response to therapy intervention, although scant clinical evidence exists comparing treatments between these groups and their counterparts. The presence of *MEN1* mutations has also demonstrated improved progression-free survival to multiple therapy classes in patients with NETs (*32*, *33*). Similarly, belzutifan efficacy is linked to *VHL* mutation status, which was only found in PNETs in our cohort. Based on clinical evidence, belzutifan has been approved in patients with *VHL*-mutated PNETs (*34*). On an individual level, comparisons of PTO efficacy often fall into two categories: retrospective and prospective therapy comparisons. While none of our patients had received relevant adjuvant therapy, two patients received neoadjuvant therapy while another was a candidate for a new targeted therapy (belzutifan). For PTOs established from patient tumors, GASNET1 demonstrated resistance toward sunitinib similar to the patient progressing despite multiple cycles of therapy, whereas PNET9 demonstrated low sensitivity toward CAP:TEM. This is of critical clinical relevance: The drug resistance or low sensitivity observed in PTOs directly correlates with the aggressive clinical course and tumor progression seen in these patients. Last, the comparison of multiple tumors from the same patient could further establish effective treatment selection and identify potential sites of resistance. Although merely observational, treatment differences in tumors derived from SINET1 (four liver tumors), PNET4 (two liver tumors), PNET7 (one primary and two liver tumors), and PNET10 (four liver tumors) were detected and could guide clinical course for patients.

Although PTOs have advantages over other model types, they remain limited in simulating systemic organism-level responses and predicting the side effects of therapeutics on healthy organs. In these scenarios, more complex models such as PDXs may be necessary to accurately resemble patient cultures while keeping whole organism complexity. Current success rates are low for tissue-based PDX models in NETs; in large studies, this rate is listed as below 10%, whereas other studies only describe a single successful implantation (*35*, *36*). In cases of low tissue quantity or if in possession of stable PTOs, these could serve as another valuable cell source for implantation. There have been notable efforts to create PTOXs in other cancer types, including colon, breast, and lung (*37*). In NET research, Kawasaki *et al.* (*20*) described the implantation of a PNET PTO line that developed a PTOX, although no information was provided on its longevity. Our PTOX model developed tumor tissue through multiple passages while maintaining NET markers and grade, including low SSTR2 expression, which may be linked to the patient’s long history of NETs and prior SSTR2 neoadjuvant treatments. The maintenanance of the top 19 genes exhibiting high alteration frequencies across all the cultures—notably, *RPL11* and *SMAD4*—suggests a loss of tumor suppression within the p53 and transforming growth factor–β signaling pathways, respectively. These signatures mirror patterns observed in various malignancies and represent promising targets for novel therapeutics (*38*, *39*). Although Duchenne muscular dystrophy (*DMD*) mutations are atypical in GEP-NETs and need further study, they have been associated with gastric cancer development and are frequently associated with aggressive clinical phenotypes (*40*, *41*), a correlation potentially reflected in this patient’s case, which was characterized by rapid disease progression. While this study highlights a single culture, these findings suggest that PTOs serve as a robust screening platform for predicting engraftment success. In addition, contrary to tumor tissue, PTOs can be implanted at the researcher’s preference, allowing for better experimental planning and resource allocation.

Despite our success in establishing expanded cultures of GEP-NET PTOs, there are still areas for improvement. Our study only examined organoids propagated until passage 6. While this is further than prior studies, additional characterization is required to ensure the stability of organoids, both in terms of cell populations in long-term cultures and genomics for our populations surpassing passage 6. We also envisage improvement for G1 PTO cultures as none of the cultures in our study were able to persist past passage 4 before growth ceased. As these cells grow more slowly, ensuring tumor cell purity and preventing growth of other cell populations including fibroblasts remains a top priority. While we were able to compare multiple clinical backgrounds for PTO growth and therapy response, further investigation in larger datasets is recommended. For example, due to the short treatment duration, we may not be capturing the development of resistant tumor cell populations. Last, while some genetic mutations were preserved, others associated with lower-grade tumors, such as *DAXX*, were lost in subsequent passages. It is likely that these gene mutations were selected against in long-term culture. We also observed new mutations within both our PTO and PTOX cultures. Populations with these mutations may exist below our calling thresholds for sequencing, with positive selection increasing their numbers toward detection. As GEP-NETs demonstrate low overall numbers of mutations, sequencing can often identify populations with low prevalence with unknown roles in tumor behavior. Further work is required to investigate these issues.

In summary, we have demonstrated the robust feasibility of culturing patient-derived GEP-NET tumor organoids long-term, enabling highly successful therapy screening. Future optimization efforts should therefore prioritize the long-term, reproducible growth of low-grade tumors.

## MATERIALS AND METHODS

### Patient tissue acquisition

Specimens were procured under an Institutional Review Board (IRB) protocol approved by the National Institutes of Health (NIH) IRB (09-C-0242). Informed consent was obtained from all patients with GEP-NET undergoing surgical procedures. The specimens were placed in RPMI and transferred to the laboratory within 30 min postsurgery.

### Tissue dissociation

Tumor dissociation and organoid formation were performed immediately postsurgery. Tumors were rinsed twice in a wash solution of Dulbecco’s Phosphate-Buffered Saline (DPBS) with 1× antibiotic-antimycotic (15240062, Thermo Fisher Scientific, Waltham, MA, USA). Tumors were minced finely, and necrotic tissues were discarded. Tissue was placed into gentleMACS C Tubes (130-093-237, Miltenyi Biotec, Gaithersburg, MD, USA), and tumor dissociation was performed using the Tumor Dissociation Kit human (130-095-929) on a gentleMACS Octo Dissociator with Heaters (130-096-427, Miltenyi Biotec), according to the manufacturer’s instructions. After the tumor was dissociated, an equal volume of cold RPMI 1640 medium was added to quench enzymatic activity, and the tumor suspension was filtered through MACS SmartStrainers (70 μm) (130-098-462) to remove undigested tissue, with the flow-through centrifuged. Ammonium-Chloride-Potassium (ACK) lysis buffer (118-156-721, Quality Biological, Gaithersburg, MD, USA) was used to lyse red blood cells according to the manufacturer’s instructions. Cells were counted using a Cellometer Auto 1000 (Nexcelom, Lawrence, MA, USA), and live cell counts were used to generate organoids.

### Organoid culture

NET organoid culture medium was developed for the growth of organoids and contained the following: advanced Dulbecco’s modified Eagle’s medium/F12 (12634010, Gibco), N2 supplement (17502001, Gibco), B27 supplement (17504044, Gibco), 1% penicillin/streptomycin, Primocin (0.1 mg/ml; ant-pm-2, InvivoGen, San Diego, CA), 1× GlutaMAX (35050061, Gibco), 1% Hepes (15630130, Gibco), 1.25 mM *N*-acetyl cysteine (A7250, Sigma-Aldrich, St. Louis, MO, USA), 10 mM nicotinamide (N3376, Sigma-Aldrich), 500 nM A-83-01 (100-1041, STEMCELL Technologies, Cambridge, MA, USA), 10 μM SB202190 (S1077, SelleckChem, Houston, TX, USA), epidermal growth factor (50 ng/ml; AF-100-15, PeproTech, Cranbury, NJ, USA), recombinant human IGF-1 (100 ng/m; 100-11, PeproTech), 10 nM gastrin I (3006, Tocris), 1 μM prostaglandin E2 (S3003, SelleckChem), and fibroblast growth factor–basic (1 ng/ml; 100-18B, PeproTech). During the first week of culture, 10 μM Y27632 (S1049, SelleckChem) was used and then removed from the following NET organoid formulations.

To form organoids, 10 million viable cells/ml were resuspended in a 50% growth factor–reduced Lactate Dehydrogenase-Elevating Virus (LDEV) Matrigel (356231, Corning) and 50% NET organoid culture medium. Long-term cultures of organoids were generated by adding 10-μl droplets of viable tumor cells suspension to each well of a non–tissue culture–coated 12-well plate, with five droplets added per well. Subsequently, plates were incubated at 37°C for 1 hour followed by the addition of 1 ml of NET organoid medium to each well. For therapeutic screening, cultures of organoids were formed by adding 1-μl droplets of viable tumor cells suspension to each well of a 96-well plate, followed by a 30-min incubation at 37°C. Following solidification of the gel, 200 μl of NET organoid medium was added to each well of the therapeutic screening plate. Complete medium changes were performed weekly.

Long-term cultures of organoids were propagated until organoid cell clusters developed >100 μm or until significant overgrowth was present. At this point, organoids were prepared for passaging by collecting NET medium, followed by a phosphate-buffered saline (PBS) wash that was combined with the NET organoid medium. This solution was centrifuged at 300*g* for 5 min to collect any loose organoids. An organoid dissociation solution was prepared by diluting dispase (5 U/ml; 07913, STEMCELL Technologies) with RPMI to obtain a solution (1 U/ml). Organoid dissociation solution was added to each well of the long-term plates, with solution pipetted up and down to dislodge matrigel domes. The centrifuged wash solution was resuspended in dissociation solution and added to the plates, which were placed at 37°C for 30 min. Afterward, organoids were removed from the wells, which was followed by another RPMI wash to remove remaining cells and debris. The dissociated matrigel and organoid solution was centrifuged at 300*g* for 5 min. The pellet was then incubated in 5 ml of accutase (07920, STEMCELL Technologies) to break up organoid structures further. Organoids were passaged at a 1 to 2.5 ratio onto new plates to continue propagation as listed above. Organoids were frozen at a ratio of one-half plate per cryovial in Recovery Cell Culture Freezing Medium (126480010, Thermo Fisher Scientific). For repeat therapy screens at passage 3 for G1 and passage 6 for G2/G3, frozen vials were thawed at 37°C, and viable cells were counted and replated as described above.

### Immunohistochemistry

Matrigel domes containing organoids at passages 0, 3, and/or 6 were removed from long-term culture plates and placed into separate tubes. Organoids were washed twice with PBS and fixed in 4% paraformaldehyde for 2 hours. Paraformaldehyde solution was removed, and organoids were washed with sterile water. The water was replaced with 70% ethanol, after which organoids were dehydrated through graded alcohols, cleared in xylene, and then infiltrated with paraffin for preparing formalin-fixed paraffin-embedded organoid blocks. These blocks were sectioned on a microtome at 5 μm and placed onto slides. The sections were stained with hematoxylin and eosin, ki67 (ab16667, Abcam, at 1:200 with pH 6 citrate buffer antigen retrieval), chromogranin A (NB120-151060, Novus, at 1:2000 with pH 6 citrate buffer antigen retrieval), synaptophysin (ab32127, Abcam, at 1:1000 with pH 6 citrate buffer antigen retrieval), and SSTR2 (ab134152, Abcam; 1:25). Slides were reviewed by an expert pathologist for NET morphology and grading.

ki67-positive cells as an index of cell proliferation was calculated as a percentage of cell nuclei with positive chromogenic expression over total cell nuclei. A minimum of 500 nuclei and three high-powered fields were counted for each organoid line.

### Sample preparation and sequencing for whole-genome sequencing

At each organoid culture passage (0, 3, and 6), Matrigel domes were removed as described above. Organoid DNA and DNA isolated from patient blood samples was isolated using the DNeasy Blood and Tissue Kit (69504, QIAGEN) according to the manufacturer’s instructions. Libraries were prepared using the TruSeq Nano DNA Prep kit. The whole-genome sequencing samples were pooled and sequenced on NovaSeq X Plus 10B and 1.5B flow cells using paired-end sequencing mode. Sequencing reads were mapped, and somatic variants were called using Illumina DRAGEN v4.2.4 to the hg38 reference genome. Percent total mapping against the reference genome was about 97%, and uniquely mapped reads are above 75%. The percentage of nonduplicate reads was determined by measuring the percentage of unique fragments in the mapped reads using MarkDuplicate utility. Percent duplicated reads are between 11 to 21%. The mean mapped sequencing depth coverage (after alignment and marking duplicates) was between 29× and 87×.

### Somatic variant analysis and visualization

Somatic variant analysis was performed using paired organoids (passage 0 versus either passage 3 or 6), depending on the last passage number (3 or 6) when the PTO culture was stopped. Somatic variants were annotated using Variant Effect Predictor (VEP) to generate Mutation Annotation Format files for downstream analysis. The R package maftools (v2.20) was used, and variants passing quality filters were retained for downstream analysis. TMB was calculated, and clinical variant annotations were added using CLINVAR (RRID:SCR_006196) database. Custom scripts were used to generate oncoplots using the ComplexHeatmap (v2.20) R package.

### Clonal evolution analysis

Subclonal populations were identified using the SuperFreq (v1.6) R package, which integrates somatic variant calling, copy number analysis, and mutation clustering (*42*). Copy number variants (CNVs) were identified by using the read depth and B-allele frequencies. Previously identified somatic mutations and CNVs were clustered into subclones on the basis of their allele frequencies across samples, using a Bayesian framework to estimate subclonal structures. River plots were generated, which groups CNV profiles and mutation clusters to allow for clonal tracking across time points per sample.

### Pharmacological treatment of PTOs

All PTO therapy treatments were performed for 96 hours (table S5). Cabozantinib (S1119, SelleckChem), pazopanib (S3012, SelleckChem), everolimus (S1120, SelleckChem), sunitinib (S7781, SelleckChem), capecitabine (S1156, SelleckChem), temozolomide (S1237, SelleckChem), cisplatin (S1166, SelleckChem), etoposide (S1225, SelleckChem), belzutifan (S8886, SelleckChem), dabrafenib (S2807, SelleckChem), trametinib (S2673, SelleckChem), streptozotocin (S1312, SelleckChem), and doxorubicin (E2516, SelleckChem) stock solutions were prepared. Fresh solutions were prepared every 3 months. Three treatments were used in combinations translated from previous clinical regimens: CAP:TEM (2:1 ratio), dabrafenib and trametinib (20:1), and cisplatin with etoposide (1:1) (*43*–*45*).

At day 10 of PTO culture in 96-well plates, medium was replaced with 100 of fresh medium and 100 μl of treatment solution containing drug. For control wells, 100 μl of 0.5% dimethyl sulfoxide was added. Dosing was performed according to table S3, ending with 200 μl of medium in each well. Following 96 hours of treatment incubation, LIVE/DEAD imaging (L3224, Invitrogen) was performed, followed by CellTiter Glo 3D assay (G9683, Promega, Madison, WI) to determine cell viability. Values were normalized to the control for further analysis.

### Implantation of PTOs into NSG mice

All mice used were 8- to 12-week-old immune-compromised NSG (the Jackson Laboratory, stock strain no. 005557) mice, male or female, under the approved by National Cancer Institute–Frederick Animal Care and Use Committee animal study protocol no. 23-044

At passage 6, PTOs (equivalent to 500,000 cells) were resuspended into 300 μl of 1:1 Matrigel/serum-free medium and implanted subcutaneously into the flanks of anesthetized (1 to 2.5% isoflurane inhalation anesthesia) mice. All work was performed in a class II type B2 biological safety cabinet. One to two drops of 0.25% bupivacaine have been applied to the incision site, before closure, to alleviate postoperative pain. Wound clips were removed 7 to 10 days postimplantation. Mice were monitored daily to look for signs of tumorigenesis. Body weights were recorded, and caliper measurements of subcutaneous tumors were performed weekly. Once mouse tumors approached >2000 mm^3^, recipient animals reached 1 year following implantation, or mouse behavior or general health changed, mice were humanely euthanized by CO_2_ asphyxia, and tissues were collected for biomarker analyses, banking by live cryopreservation, and for serial implantation into new recipient mice using 1- to 5-mm^3^ tissue fragments.

### Sample identity concordance

Sample identity concordance was performed using two independent analyses to confirm PTOX cellular origin from a single tissue source. First, cell line authentication was performed by IDEXX BioAnalytics (Columbia, MO, USA) using the CellCheck service: Samples were authenticated using STR profiling. Human samples were analyzed using 16 STR loci and amelogenin to confirm identity. Interspecies contamination was screened via multiplex PCR. Samples were compared to the original tumor specimen (GasNET1 patient). Second, Picard CrosscheckFingerprints (GATK v4.6.2.0) with the GRCh38 haplotype database (Broad Institute) was used across the patient-matched germline blood sample (GasNET1 patient), three GASNET organoid passages (p0_GasNET1a, p3_GasNET1a, and p6_GasNET1a), and two PDX passages (p0_GasNET1a_PTOX and p1_GasNET1a_PTOX) to assess patient cell of origin. The germline blood BAM (bag of marbles) was used directly as the identity anchor. PDX BAMs were prefiltered to Mapping Quality (MAPQ) of ≥30 primary alignments before fingerprinting to reduce mouse stromal read contamination. Tumor-aware LOD scores were computed to account for potential loss of heterozygosity in tumor-derived samples. All pairwise tumor-aware LOD scores exceeded 72, confirming common donor origin across all passages. Results were clustered using ClusterCrosscheckMetrics (LOD threshold of 5); all six samples were assigned to a single cluster.

### Statistics

For therapeutic efficacy calculations, GraphPad Prism10 (RRID:SCR_002798) was used to calculate dose-response IC_50_ curves from treatment responses for all GEP-NET organoids, for all tumor grades, and for each NET subtype. To assess treatment responses against the group, IC_50_ for each PTO and treatment was divided against the GEP-NET average for the treatment. For drug response visualization, a heatmap was created with dose response represented as −log_2_(ratio). Values were normalized, with maximum log_2_(difference) of 5 used in graphing treatment response and in comparing tumor treatment response between groups. Comparisons between treatment groups were performed using Student’s *t* test (two-sample, two-tailed, and unpaired) where applicable.
