# Supplementary material for: Expanded GEP-NET organoid culture for personalized therapy evaluation
Source: Sci Adv. 2026 Jul 24;12(30):eaea4296. doi: 10.1126/sciadv.aea4296 (PMC13398536; doi:10.1126/sciadv.aea4296)
Supplement: Supplementary file 1 — Figs. S1 to S4 Legends for tables S1 and S2 Tables S3 to S5 Legends for data S1 to S5 [file sciadv.aea4296_sm.pdf]

Science Advances
AAAS

Supplementary Materials for

Expanded GEP-NET organoid culture for personalized therapy evaluation

Steven D. Forsythe *et al.*

Corresponding author: Samira M. Sadowski, samira.sadowski@nih.gov

Sci. Adv. **12**, eaea4296 (2026)
DOI: 10.1126/sciadv.aea4296

The PDF file includes:

Figs. S1 to S4
Legends for tables S1 and S2
Tables S3 to S5
Legends for data S1 to S5

Other Supplementary Material for this manuscript includes the following:

Tables S1 and S2
Data S1 to S5

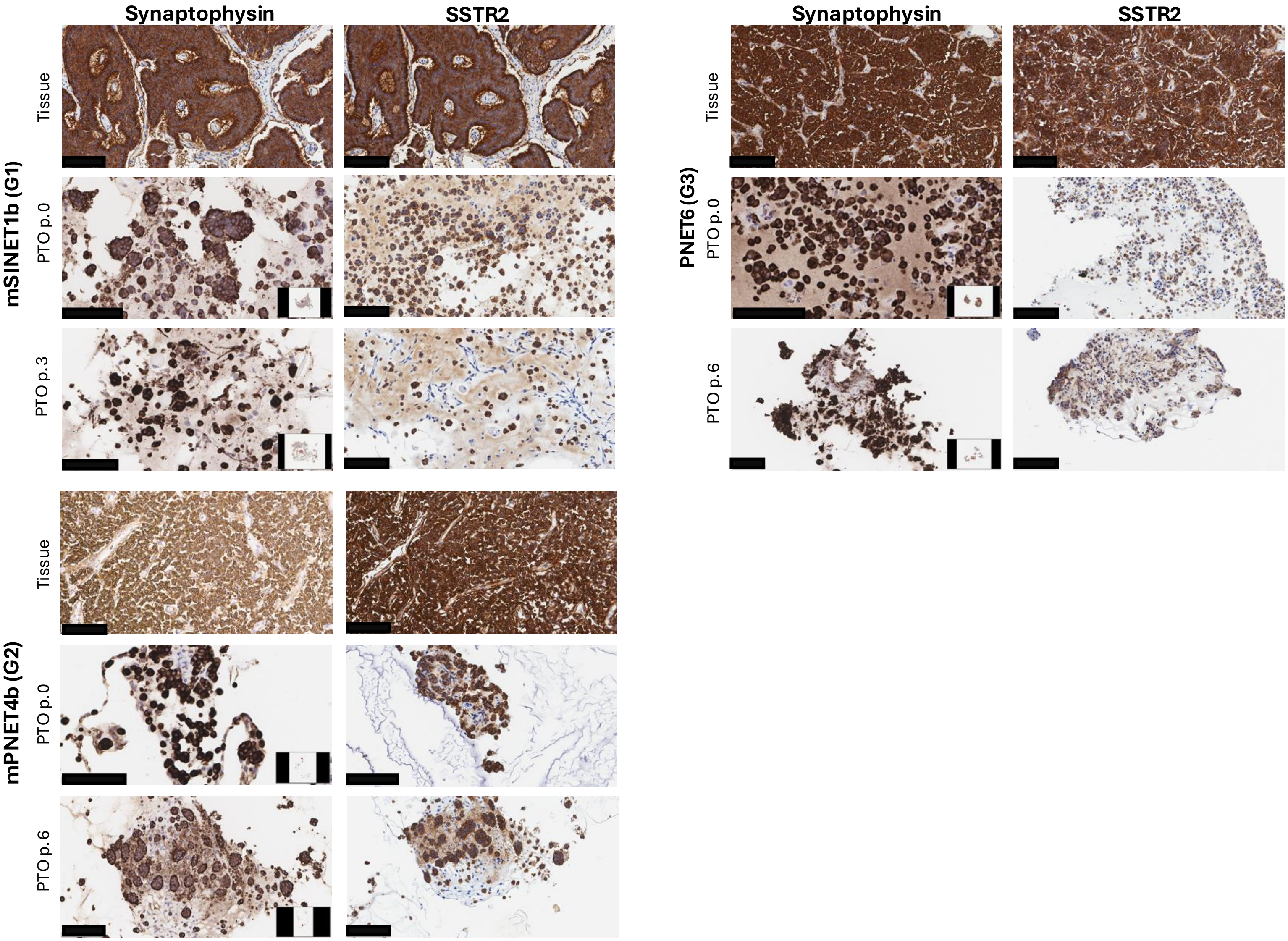

Fig S1. GEP-NET organoids maintain expression of neuroendocrine tumor marker synaptophysin and surface membrane SSTR2. Representative samples of all three grades. Scale bar=100 μm.

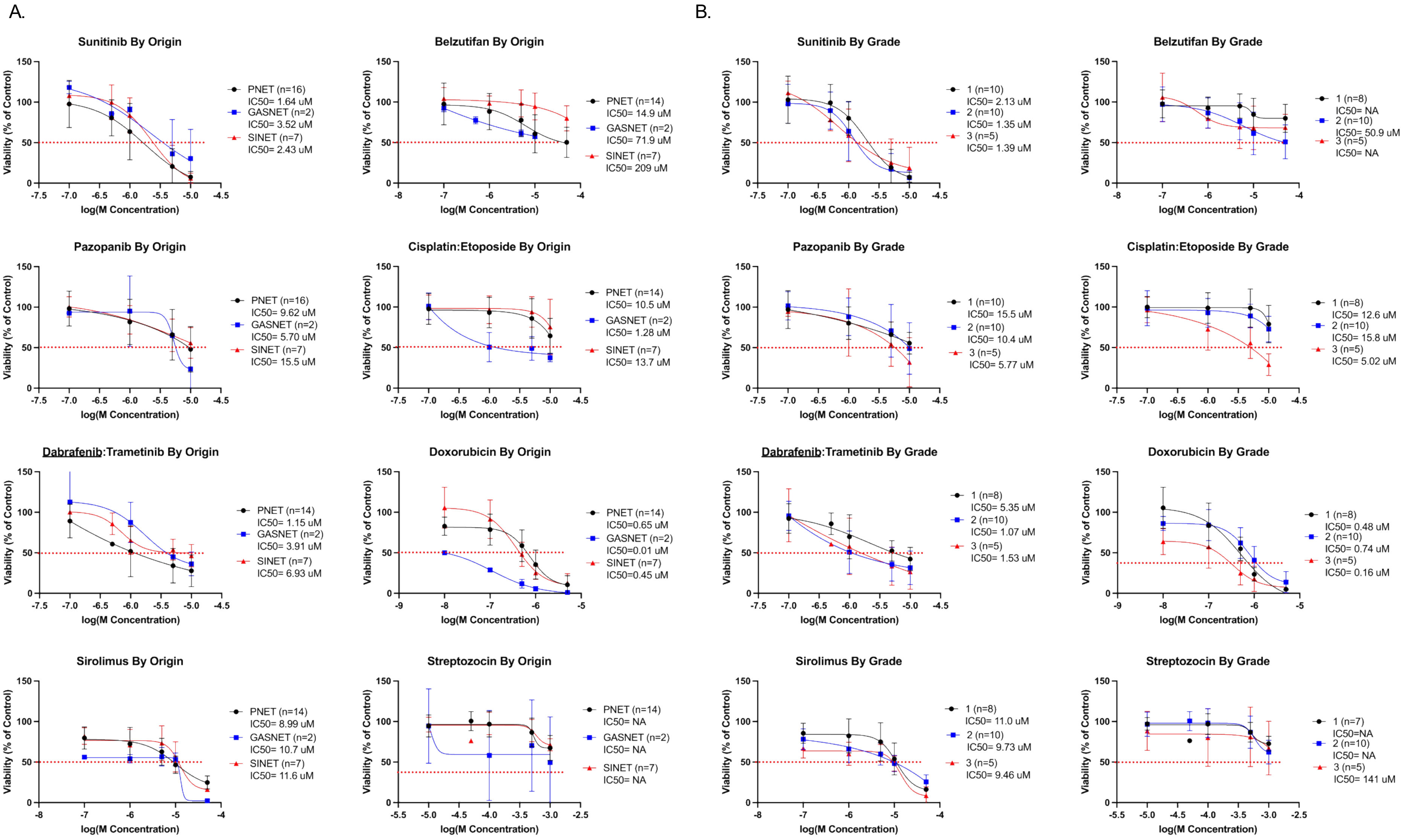

Fig S2. **GEP-NET PTO treatment summary for sunitinib, pazopanib, dabrafenib:trametinib, sirolimus, belzutifan, cisplatin:etoposide, doxorubicin, and streptozocin.** A. GEP-NET PTO treatment dose response curves by origin B. GEP-NET PTO treatment dose response curves by grade. Each point consists of biological replicates.

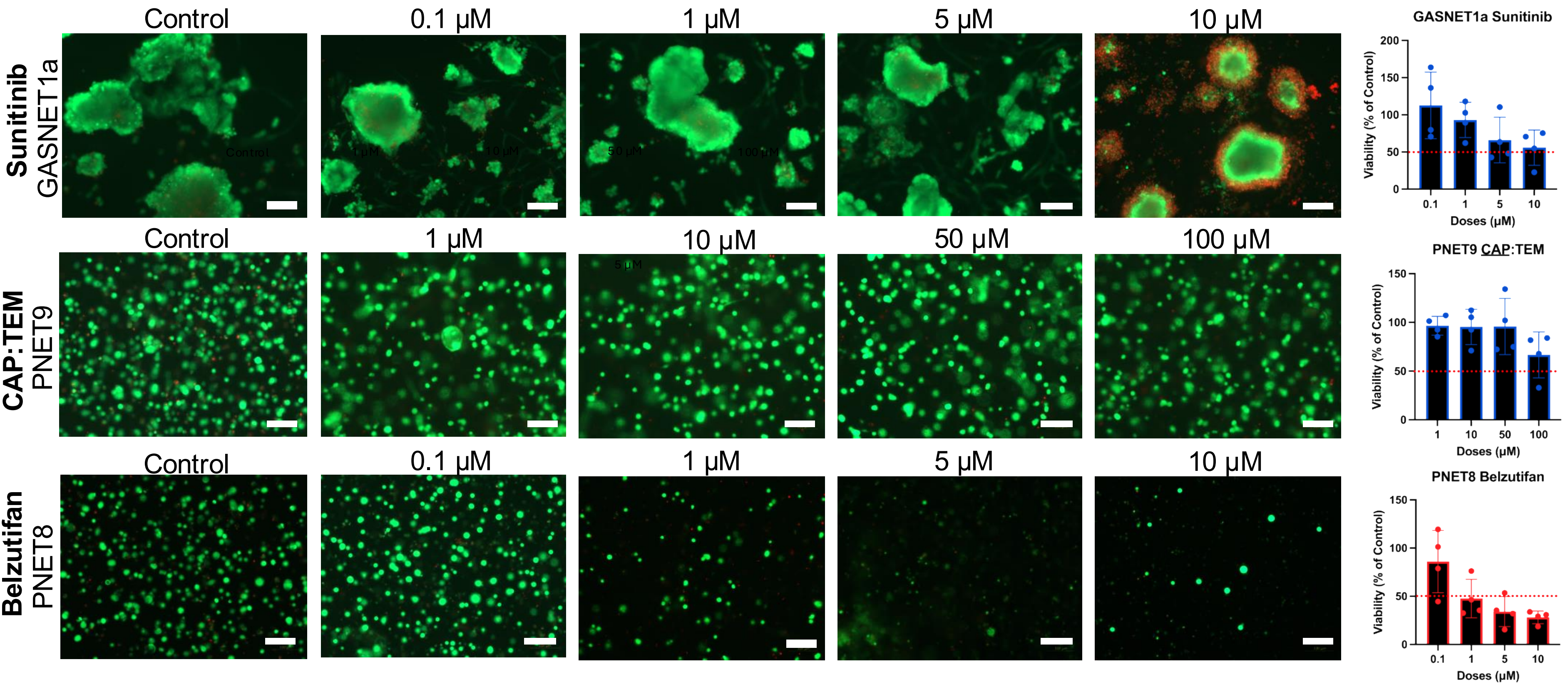

Fig S3. LIVE/DEAD panels for GEP-NET PTOs derived from patients with non-SSTR targeted neoadjuvant treatments. Scale bar= 100 µm. n=4 technical replicates.

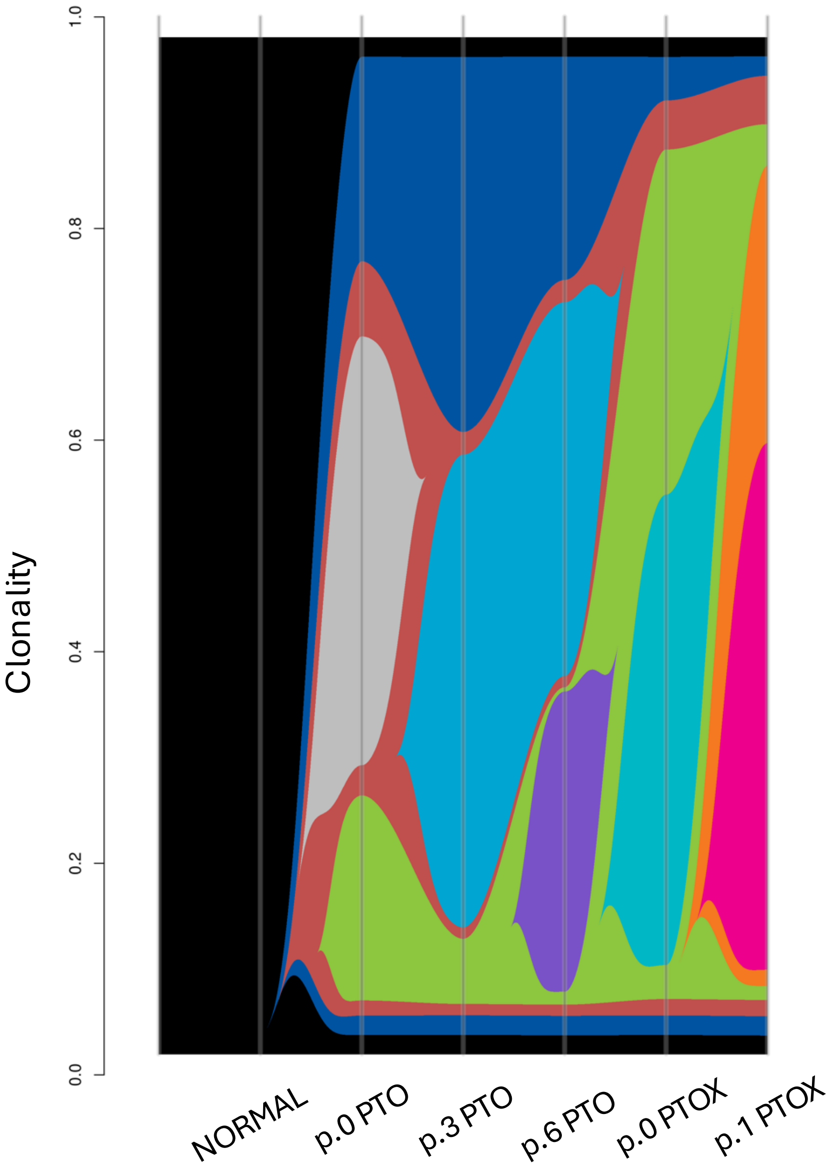

Fig S4. **River Plot for GASNET1A PTOs and PTOX**. River plot demonstrating clonal population evolution of tumor cells from the original passage 0 organoid to passage 1 PTOX.

Sample	Normal	p0 PTO	p3 PTO	p6 PTO	p0 PTOX	p1 PTOX
Normal	98.74	74.96	77.79	74.10	73.68	72.35
p0 PTO	74.96	102.46	101.85	102.45	97.19	99.87
p3 PTO	77.79	101.85	101.68	101.84	96.73	99.26
p6 PTO	74.10	102.45	101.84	102.44	97.18	99.86
p0 PTOX	73.68	97.19	96.73	97.18	100.81	95.23
p1 PTOX	72.35	99.87	99.26	99.86	95.23	102.65

Table S3. Sample identity concordance across all GasNET1. Tumor-aware LOD scores account for potential loss of heterozygosity in tumor-derived samples. A positive LOD score indicates evidence of shared donor origin; scores > +5 are considered confident matches.

Marker	GasNET1a Tumor	GasNET1a PTO p.6	GasNET1a PTOX p.0	GasNET1a PTOX p.1
**AMEL**	X, Y	X, Y	X, Y	X, Y
**CSF1PO**	10, 11	10, 11	10, 11	10, 11
**D13S317**	12, 13	12, 13	12, 13	12, 13
**D16S539**	11, 13	11, 13	11, 13	11, 13
**D18S51**	13	13	13	13
**D21S11**	27, 29	27, 29	27, 29	27, 29
**D3S1358**	14, 16	14, 16	14, 16	14, 16
**D5S818**	10, 12	10, 12	10, 12	10, 12
**D7S820**	9, 10	9, 10	9, 10	9, 10
**D8S1179**	10, 14	10, 14	10, 14	10, 14
**FGA**	20	20	20	20
**Penta D**	9, 14	9, 14	9, 14	9, 14
**Penta E**	7, 11	7, 11	7, 11	7, 11
**TH01**	8	8	8	8
**TPOX**	11, 12	11, 12	11, 12	11, 12
**vWA**	17, 19	17, 19	17, 19	17, 19
**Identity Match***	N/A#	100%	100%	100%

Table S4- IDEXX STR 16- Marker analysis confirms single patient origin of PTO and PTOX cultures. *For human samples, an identity matching score above 80% indicates the sample is consistent with the tissue of origin. Both PTOX samples tested positive for mouse and human DNA, an expected finding with transplanted tumors in mice. N/A # in the table indicates profile data is not available for comparison purposes for this sample. The genetic profile for this primary patient sample was compared to the cell line genetic profiles available in the DSMZ STR database and did not match any other reported profiles in the DSMZ database.

Therapy Concentration (μM)					
Everolimus	0.1	1	5	10	50
Cabozantinib	0.1	1	5	10	50
Pazopanib	0.1	1	5	10	50
Sunitinb	0.1	0.5	1	5	10
Belzutifan	0.1	1	5	10	50
Dabrafenib:Trametinib	0.1:0.005	0.5:0.01	1:0.05	5:0.1	10:0.5
Capecitabine:Temozolomide	0.1:0.05	1:0.5	10:5	50:25	100:50
Streptozotocin	10	50	100	500	1000
Cisplatin:Etoposide	0.1:0.1	1:1	5:5	10:10	50:50
Doxorubicin	0.01	0.1	0.5	1	5

Table S5. Therapy concentrations for patient-derived tumor organoid therapy screening.

Data present in separate files

Table S1 Patient and PTO Dataset. GEP-NET patient clinical information and patient tumor organoid summary. SBRT- Stereotactic Body Radiation, NA=Not available. Prior NET reflects previous surgery to remove a NET.

Table S2 PTO Days to Passage. GEP-NET patient tumor organoid passaging data. P= Primary. M= Metastasis

Data S1. ki67 Quantification

Data S2. River Plot Populations

Data S3. PTO Therapy Screening Celltiter Glo Value

Data S4. PTO Repeat Passaging Celltiter Glo Therapy Values

Data S5. PTOX Growth Data
